# Causal associations of particulate matter 2.5 and cardiovascular disease: A two-sample mendelian randomization study

**DOI:** 10.1371/journal.pone.0301823

**Published:** 2024-04-05

**Authors:** Ye Cao, Yi Feng, Nan Xia, Jiancheng Zhang

**Affiliations:** 1 Department of Cardiology, Fujian Provincial Hospital, Shengli Clinical Medical College of Fujian Medical University, Fuzhou, Fujian, P. R. China; 2 Department of Cardiology, Renmin Hospital, Hubei University of Medicine, Shiyan, Hubei, P. R. China; Kanazawa University, JAPAN

## Abstract

**Background:**

According to epidemiological studies, particulate matter 2.5 (PM2.5) is a significant contributor to cardiovascular disease (CVD). However, making causal inferences is difficult due to the methodological constraints of observational studies. In this study, we used two-sample Mendelian randomization (MR) to examine the causal relationship between PM 2.5 and the risk of CVD.

**Methods:**

Genome-wide association study (GWAS) statistics for PM2.5 and CVD were collected from the FinnGen and UK Biobanks. Mendelian randomization analyses were applied to explore the causal effects of PM2.5 on CVD by selecting single-nucleotide polymorphisms(SNP) as instrumental variables.

**Results:**

The results revealed that a causal effect was observed between PM2.5 and coronary artery disease(IVW: OR 2.06, 95% CI 1.35, 3.14), and hypertension(IVW: OR 1.07, 95% CI 1.03, 1.12). On the contrary, no causal effect was observed between PM2.5 and myocardial infarction(IVW: OR 0.73, 95% CI 0.44, 1.22), heart failure(IVW: OR 1.54, 95% CI 0.96, 2.47), atrial fibrillation(IVW: OR 1.03, 95% CI 0.71, 1.48), and ischemic stroke (IS)(IVW: OR 0.98, 95% CI 0.54, 1.77).

**Conclusion:**

We discovered that there is a causal link between PM2.5 and coronary artery disease and hypertension in the European population, using MR methods. Our discovery may have the significance of public hygiene to improve the understanding of air quality and CVD risk.

## 1 Introduction

Air pollution is a complicated combination of particulate matter (PM) and gaseous contaminants. Although the relative hazards associated with air pollution are lower than those associated with more established risk factors (such as hyperlipidemia and cigarette use), it nevertheless constitutes a danger to public health since it affects billions of people every day without their consent [1]. PM is the major component of air pollution and is made up of a variety of aerosols with different sizes and compositions, such as metals, carbon dioxide, organic species, and inorganic nitrates and sulfates [2, 3]. There is broad scientific agreement that among size portions, PM2.5 is the most firmly established to have negative health consequences and is responsible for the largest worldwide public health burden [4].

Although exposure to PM2.5 has been linked to a variety of health issues, cardiovascular disease(CVD) is the primary cause of PM2.5-related mortality [5, 6]. Moreover, PM2.5 has been identified as a significant risk factor for cardiovascular morbidity and death by health organizations throughout the world, including the American Heart Association and the European Society of Cardiology [7, 8]. The mechanism research validated that exposure to PM2.5 will impact systemic inflammation, oxidative stress, and the autonomic nervous system. The endothelial, endocrine, and thrombosis pathways will be affected by these consequences, which will cause hypertension, arrhythmia, and artery stiffness, and eventually lead to CVDs [9]. Numerous epidemiological studies and meta-analyses have established the link between exposure to PM2.5 and an increased risk of CVDs, including coronary artery disease(CAD), myocardial infarction(MI), heart failure(HF), atrial fibrillation(AF), ischemic stroke(IS), and hypertension (HTN) [10].

However, the above association is mainly based on epidemiological research. Although the quality and amount of evidence generated in the recent past have improved significantly, observational research is essentially affected by residual confusion and reversal causation. Additionally, no research has yet looked at the combined impact of PM2.5 and genetic polymorphisms on the risk of CVD. Prior research indicates that genetic polymorphism should be taken into account when exploring the effects of pollutants on various physiological and immunological processes in humans, especially in cardiovascular diseases and respiratory diseases [11, 12]. For instance, a panel study revealed that individuals with the glutathione S-transferase mu 1 genotype are more vulnerable to air pollution [12].

Mendelian randomization (MR) is a unique method for examining the causal relationship between risk factors and outcomes that employs genetic variations as instrumental variables (IVs), and this approach is extensively utilized in the study of CVD [13, 14]. Because genetic variations are assigned at random before illness starts, MR analysis might eliminate confounding variables and reverse causation, allowing researchers to find more causal drivers of a certain outcome [15]. In our study, a two-sample MR research was used to explore the potential causality between PM2.5 and CVD risk with the use of extensive genome-wide association study (GWAS) data.

## 2 Materials and methods

### 2.1 Study design

This study adhered to the STROBE-MR (Strengthening the Reporting of Observational Studies in Epidemiology Using Mendelian Randomization) guidelines [16], and the STORBE-MR checklist is provided in **S1 Table**. Only when the following MR study assumptions are met may a convincing conclusion be reached. First, the exposure (PM2.5) and the genetic IVs should be highly related. Second, the possible confounders such as body mass index(BMI), lipoproteins (total cholesterol, triglycerides, low-density lipoprotein, high-density lipoprotein), smoking status, diabetes, blood pressure, and others have nothing to do with the genetic IVs. Third, the genetic IVs only affect the outcome (CVD) via PM2.5 [17, 18].

### 2.2 Data sources

In this study, summary-level results from GWAS were utilized. GWAS summary statistics for PM2.5 included a total of 423,796 European participants with 9,851,867 SNPs. The outcomes include CAD, MI, HF, AF, IS, and HTN. For the same circumstance, we acquired summary-level data for the outcome events from the GWAS database. The basic information about the GWAS database of the exposure and outcomes can be seen in **Table 1**.

**Table 1:** Basic information about the GWAS database of the exposure and outcomes.

Trait	GWAS-ID	Year	Population	Number of SNPs	Sample size
Particulate matter air pollution (pm2.5)	ukb-b-10817	2018	European	9,851,867	423,796
Coronary artery disease	ebi-a-GCST005195	2017	European	7,934,254	ncase: 122,733
					ncontrol: 424,528
Myocardial infarction	finn-b-I9_MI	2021	European	16,380,433	ncase:12,801
					ncontrol:187,840
Heart failure	ebi-a-GCST009541	2020	European	7,773,021	ncase:47,309
					ncontrol:930,014
Atrial fibrillation	ebi-a-GCST006414	2018	European	33,519,037	ncase:60,620
					ncontrol:970,216
Ischemic stroke	ebi-a-GCST006908	2018	European	8,296,492	ncase:34,217
					ncontrol:406,111
Hypertension	ukb-b-12493	2018	European	9,851,867	ncase: 54,358
					ncontrol: 408,652

### 2.3 Selection and validation of SNPs

We used three criteria to identify independent SNPs related to PM2.5. First, SNPs with a genome-wide significance threshold of p < 5×10^−8^ were chosen. Second, paired linkage disequilibrium was employed to assess the independence of the chosen SNPs. SNPs situated at a distance of 1000 kb apart were chosen, and those in linkage disequilibrium (LD) (r^2^ <0.001) were removed [19]. Third, the F-statistic was used to confirm the power of every SNP [20]. When F-statistics were more than 10, SNPs were thought to be effective enough to counteract the effects of possible bias. Furthermore, the PhenoScanner database was used to rule out the effect of possible confounders [21].

### 2.4 Two-sample MR analysis

We employed three methods(MR egger, weighted median, inverse variance weighted) in this two-sample MR study to obtain causal relationships between PM2.5 and CVD [22]. In our MR study, the inverse variance weighted (IVW) analysis is the primary method as it produces the most convincing estimates when the IVs absent directional pleiotropy [23]. To eliminate the horizontal pleiotropy, the other two analytical methods are used. The aforementioned methods of analysis are all centered on various horizontal pleiotropy models. Thus, we may assess the reliability of our results by comparing the consistency of three different methodologies [24]. Cochran’s Q-test assessed IV heterogeneity, with a *p* < 0.05 result suggesting heterogeneity. We evaluated horizontal pleiotropy using the MR-Egger intercept test [25, 26]. Furthermore, we utilized the leave-one-out approach to determine if a single SNP disrupted the causative impact. TwoSampleMR (0.5.7) packages were used to do all statistical analyses in R software (4.2.3). A value of *p* < 0.05 was regarded as suggestive significance and associations with *p* <0.0083(Bonferroni correction *p* = 0.05/6) were considered statistically significant [27]. In addition, we assessed the power of this current MR analysis using the "mRnd" tool [28].

## 3 Results

### 3.1 The causal effect of cardiovascular disease

We got 8 SNPs related to PM2.5 based on the screening criterion. Then, using the Phenoscanner database, we eliminated the rs1537371 related to CAD and rs77205736 related to BMI. We eventually got 6 SNPs. The details of SNPs are shown in **Table 2**.

**Table 2:** SNPs of PM2.5 with statistically significant threshold.

SNP	effect_allele	other_allele	se	beta	eaf	pval	F
rs6749467	A	G	0.002183	-0.0123919	0.465814	1.37E-08	32.4
rs1372504	A	G	0.002219	0.0122914	0.374311	3.05E-08	29.9
rs12203592	T	C	0.002591	0.0216661	0.212894	6.18E-17	66.7
rs114708313	T	A	0.004478	0.024558	0.06585	4.15E-08	31.4
rs77255816	T	C	0.005728	0.0313937	0.036507	4.23E-08	29.4
rs72642437	T	C	0.019135	0.113396	0.003862	3.10E-09	41.9

**Fig 1:**
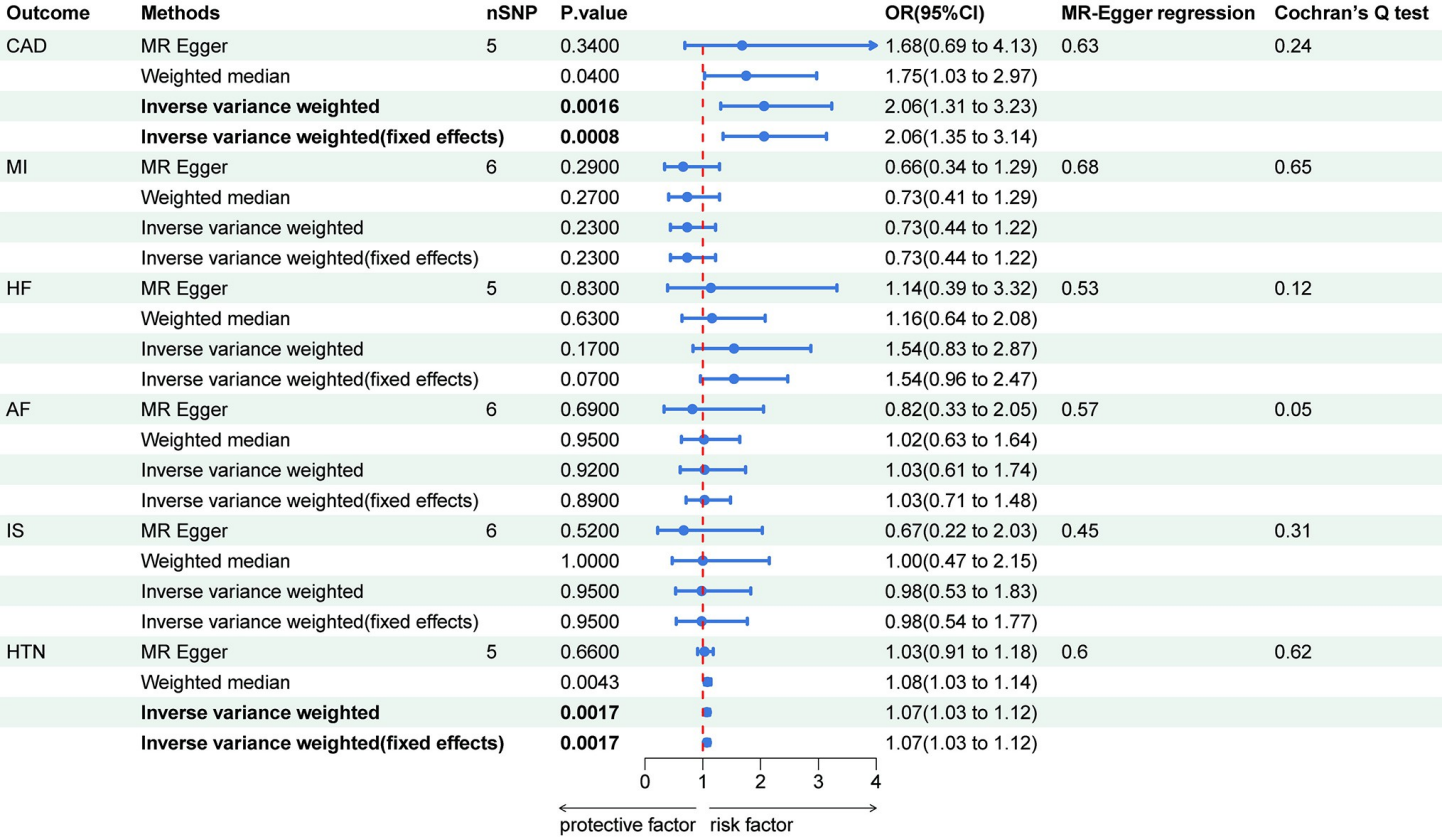
Causal associations between PM2.5 and CVD. CAD: coronary artery disease; MI: myocardial infarction; HF: heart failure; AF: atrial fibrillation; IS: ischemic stroke; HTN: hypertension.

The two-sample MR analysis using three methods for the association of PM2.5 with CVD is shown in **Fig 1 and S1 Fig**. The IVW results revealed that causal effect was observed between PM2.5 and CAD(IVW: OR 2.06, 95% CI 1.35, 3.14), and HTN(IVW: OR 1.07, 95% CI 1.03, 1.12). After multiple testing corrections, statistical differences persist. Similar results were observed using the weighted median method. On the contrary, no causal effect was observed between PM2.5 and MI(IVW: OR 0.73, 95% CI 0.44, 1.22), HF(IVW: OR 1.54, 95% CI 0.96, 2.47), AF(IVW: OR 1.03, 95% CI 0.71, 1.48), and IS(IVW: OR 0.98, 95% CI 0.54, 1.77). The MR power of PM2.5 on CAD was 1.0, and the powers of other outcomes were all less than 0.8. The MR powers are shown in **S2 Table.**

### 3.2 Sensitivity analysis validation

No significant heterogeneity was discovered by Cochran’s Q-test. Consequently, we choose to apply the fixed-effects IVW approach. Then, we used an MR-Egger intercept test and leave-one-out analysis to remove the bias caused by horizontal pleiotropy. The MR-Egger intercept test revealed no directional pleiotropy. The scatter plots of the association between PM2.5 and CVD are shown in **S2 Fig**. In the meantime, the leave-one-out analysis revealed the stability of the MR effect estimates, which are shown in **S3 Fig**.

## 4 Discussion

In this study, we found a causal relationship between PM2.5 and the risk of CAD and HTN by a two-sample MR method, while no causal relationship between PM2.5 and MI, HF, AF, and IS susceptibility.

Our research results are consistent with some previous epidemiological results. The most recent meta-analysis, published in 2023, found increased exposure to PM 2.5 levels is significantly associated with an increased risk of all-cause mortality (HR 1.08, 95% CI 1.05, 1.11), CVD(HR 1.09, 95% CI 1.00, 1.18), and CVD mortality(HR 1.12, 95% CI 1.07, 1.18) [29]. Another meta-analysis, also published in 2023, found PM 2.5 (every 10μg/m^3^ rise) increased the risk of hypertension(RR 1.14, 95% CI 1.09, 1.19), and coronary heart disease (RR 1.21, 95% CI 1.08, 1.35) [30]. In addition, the results of the other two meta-analyses did not support the notion that PM2.5 can raise the risk of AF(RR 0.89, 95% CI 0.20, 1.57) and HF(HR 1.07, 95% CI 0.72, 1.60) [31, 32]. Naturally, there is some prior research that contradicts our findings. In our investigation, there was no evidence of a causal relationship between PM2.5 and MI, AF, or IS. Nevertheless, some research has revealed that exposure to PM 2.5 increases the risk of MI [33, 34], AF [35], and IS [30, 36, 37].

The variations showed that instead of identifying a causal relationship, epidemiology study findings can be constrained by confounders and research design. It is crucial to remember that confounding factors such as different lifestyles, baseline health, socioeconomic status, additional environmental variables, and many other variables could distort this epidemiological research. Obviously, because we used summary-level data in our MR analysis, we cannot completely rule out the potential of a nonlinear causal relationship between PM2.5 and MI, AF, and IS. To investigate potential dose-response causal relationships, further MR research should be carried out using individual-level data and longitudinal designs.

Based on data from epidemiological research together with animal and toxicologic trials, the mechanism by which PM 2.5 raises the risk of CVD has been partially clarified. PM2.5 may interact with pulmonary and immunological cells, as well as neural receptors, and entering the systemic circulation can set off a chain of events that contribute to the acute and chronic impacts of CVD [38, 39]. PM2.5 may deposit on vascular endothelium, aggravating local oxidative stress and inflammation, leading to atherosclerotic plaque instability and, eventually, thrombus development [40]. PM2.5 can cause systemic inflammation and endothelial dysfunction, which can accelerate the progression of atherosclerosis [41, 42]. In addition, PM 2.5 may raise the possibility of acquiring other well-known risk factors for cardiovascular disease including diabetes, hypertension, and hyperlipidemia [43–45].

This study presents several benefits. First, this is the first study to use a two-sample MR analysis with data from the GWAS summary-level data to look at the causal link between PM2.5 and CVD. A two-sample method can lower potential confounders and reverse causality in large genomic data summaries. Second, through sensitivity analysis using several MR methods and various model assumptions, the findings were confirmed. All of the analyses indicated that the findings were solid and trustworthy.

However, this research did have certain restrictions. First, we stress that, due to the relatively small amount of PM2.5 variability that can be explained by real SNPs, our analytical power is somewhat constrained when analyzing the minimal influence of PM2.5 on CVD. Second, it is important to emphasize that our research heavily relied on pre-existing GWAS data. While there are no GWASs for distinct areas of PM2.5, it might be challenging to ascertain how different areas affect the causal relationship between PM2.5 and CVD. Third, in our analysis, the CVD GWAS statistics were limited to European groups to ensure that SNPs are not linked to any confounding factors between PM2.5 and CVD. Thus the results cannot be extrapolated to other populations. Fourth, our investigation may produce false negative results because of its inadequate powers. These results therefore need to be interpreted with caution. This suggests that to provide definitive evidence, larger-scale, specific GWAS data are required. Finally, the MR results only represent the long-term effects of PM2.5 on CVD, not the short-term effects. Therefore, more study is needed to determine the short-term impact of PM2.5 on CVD.

## 5 Conclusion

We discovered that there is a causal link between PM2.5 and CAD and HTN in the European population, using MR methods. Our discovery may have the significance of public hygiene to improve the understanding of air quality and CVD risk.

## Supporting information

S1 Fig Forest plots of the association of PM2.5 with CVD. CAD: coronary artery disease; MI: myocardial infarction; HF: heart failure; AF: atrial fibrillation; IS: ischemic stroke; HTN: hypertension.
(TIF)

S2 Fig Scatter plots of the association of PM2.5 with CVD. CAD: coronary artery disease; MI: myocardial infarction; HF: heart failure; AF: atrial fibrillation; IS: ischemic stroke; HTN: hypertension.
(TIF)

S3 Fig The leave-one-out sensitivity analysis of PM2.5 with CVD. CAD: coronary artery disease; MI: myocardial infarction; HF: heart failure; AF: atrial fibrillation; IS: ischemic stroke; HTN: hypertension.
(TIF)

S1 Table STORBE-MR checklist. (DOCX)

S2 Table The MR powers of CVD. (DOCX)
