# Correction: Causal associations of particulate matter 2.5 and cardiovascular disease: A two-sample mendelian randomization study

**DOI:** 10.1371/journal.pone.0360304

**Published:** 2026-10-07

**Authors:** Ye Cao, Yi Feng, Nan Xia, Jiancheng Zhang

There are errors in the author affiliations. The correct affiliations are as follows:

Ye Cao^1,2,3^, Yi Feng^3^, Nan Xia^3^, Jiancheng Zhang^1,2^

**1** Department of Cardiology, Shengli Clinical Medical College of Fujian Medical University, Fuzhou, Fujian, P. R. China, **2** Department of Cardiology, Fujian Provincial Hospital, Fuzhou, Fujian, P. R. China, **3** Department of Cardiology, Renmin Hospital, Hubei University of Medicine, Shiyan, Hubei, P. R. China.
