# Supplementary figures and images for: Causal associations of particulate matter 2.5 and cardiovascular disease: A two-sample mendelian randomization study
Source: PLoS One. 2024 Apr 5;19(4):e0301823. doi: 10.1371/journal.pone.0301823 (PMC10997086; doi:10.1371/journal.pone.0301823)

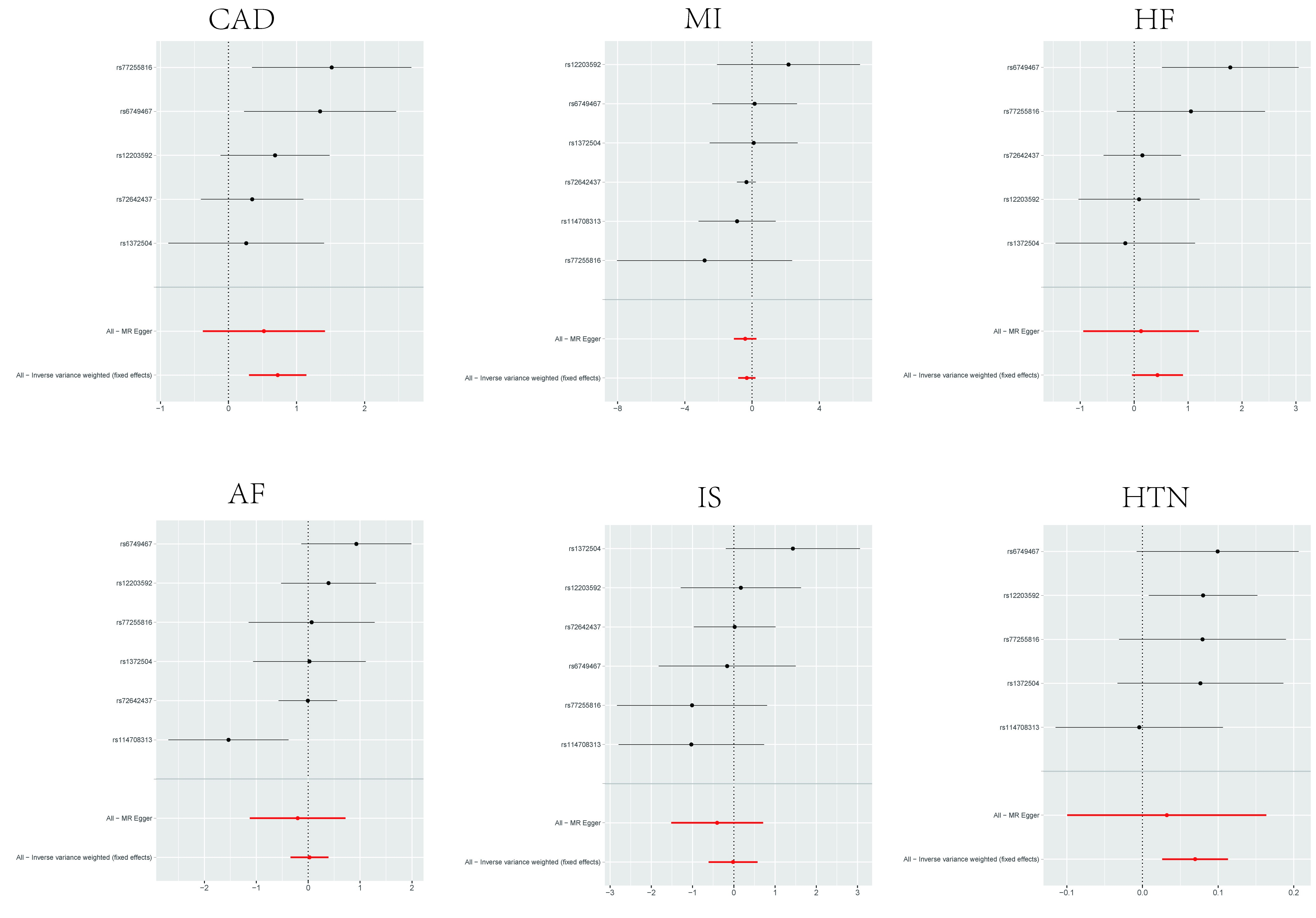

Supplement: S1 Fig — CAD: coronary artery disease; MI: myocardial infarction; HF: heart failure; AF: atrial fibrillation; IS: ischemic stroke; HTN: hypertension. (TIF) [file pone.0301823.s001.tif]

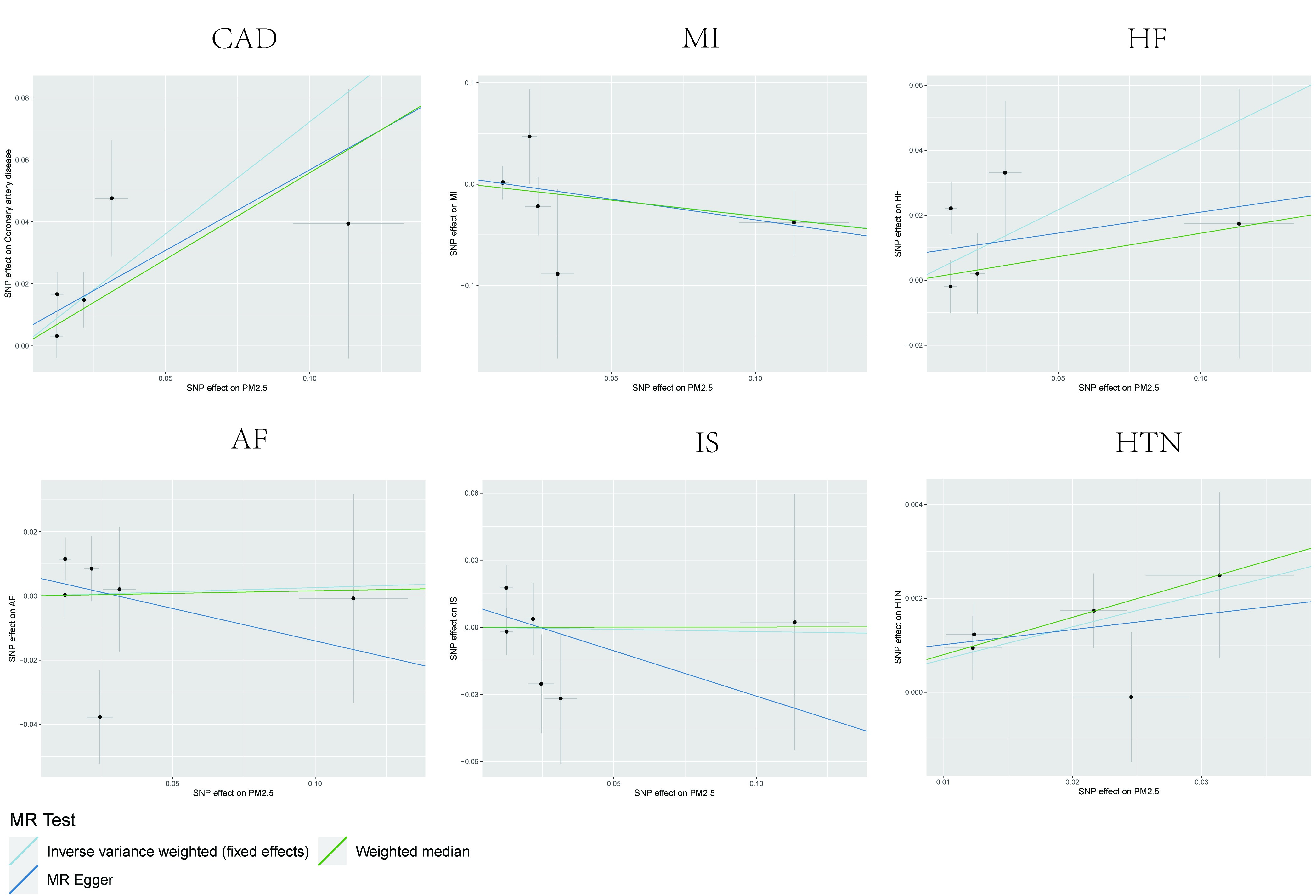

Supplement: S2 Fig — CAD: coronary artery disease; MI: myocardial infarction; HF: heart failure; AF: atrial fibrillation; IS: ischemic stroke; HTN: hypertension. (TIF) [file pone.0301823.s002.tif]

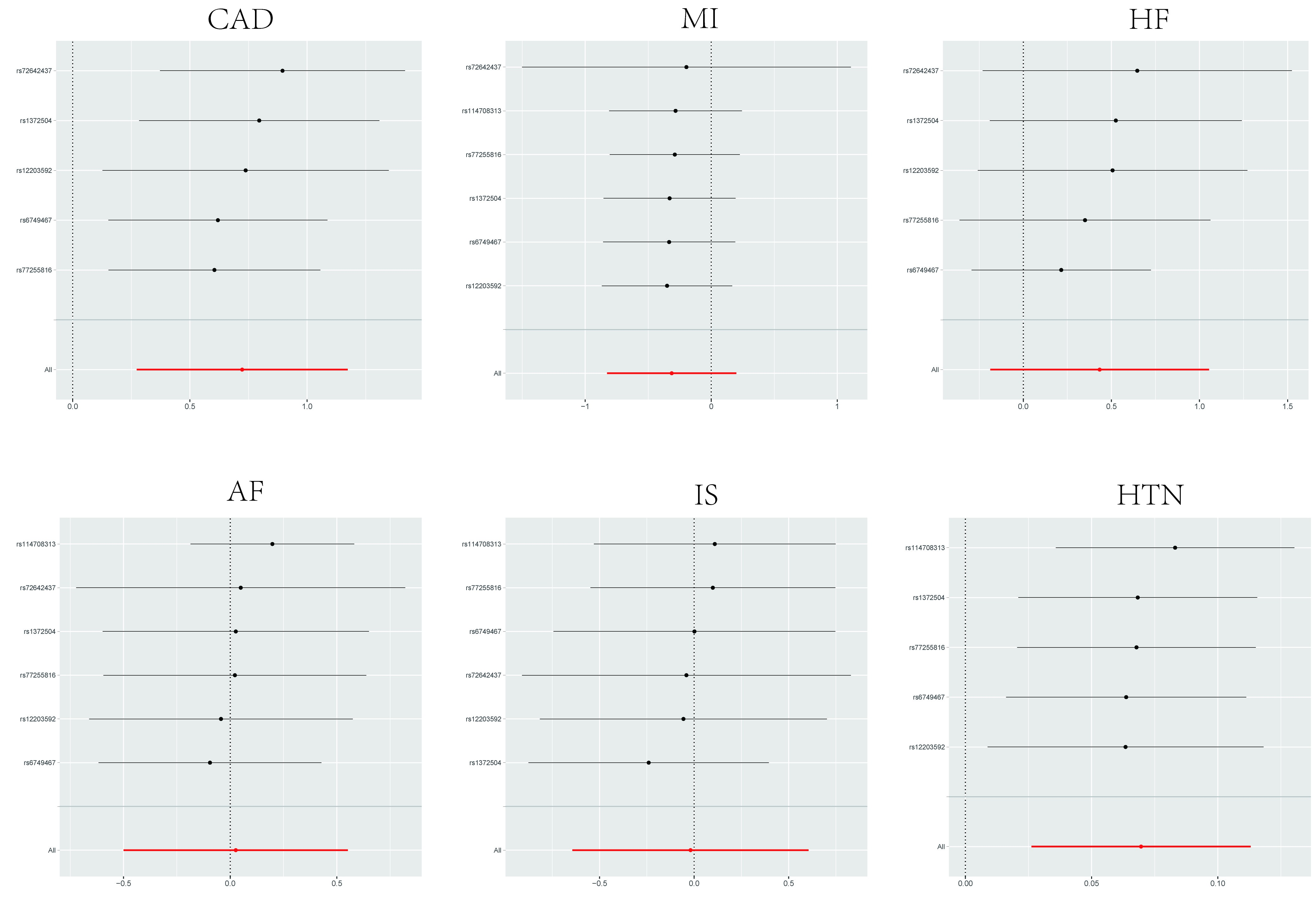

Supplement: S3 Fig — CAD: coronary artery disease; MI: myocardial infarction; HF: heart failure; AF: atrial fibrillation; IS: ischemic stroke; HTN: hypertension. (TIF) [file pone.0301823.s003.tif]
