# Supplementary material for: Causal associations of particulate matter 2.5 and cardiovascular disease: A two-sample mendelian randomization study
Source: PLoS One. 2024 Apr 5;19(4):e0301823. doi: 10.1371/journal.pone.0301823 (PMC10997086; doi:10.1371/journal.pone.0301823)
Supplement: S2 Table — (DOCX) [file pone.0301823.s005.docx]

**Table S1 The MR powers of CVD**

|  | N | K | OR | R^2^ | Power |
| --- | --- | --- | --- | --- | --- |
| Coronary artery disease | 547261 | 0.2243 | 2.06 | 0.000472778 | 1 |
| Myocardial infarction | 200641 | 0.0638 | 0.73 | 0.000546975 | 0.11 |
| Heart failure | 977323 | 0.0484 | 1.54 | 0.000472778 | 0.69 |
| Atrial fibrillation | 1030836 | 0.0588 | 1.03 | 0.000546975 | 0.05 |
| Ischemic stroke | 440328 | 0.0777 | 0.98 | 0.000546975 | 0.05 |
| Hypertension | 463010 | 0.1174 | 1.07 | 0.000448038 | 0.06 |

N: Sample size

K: Proportion of cases in the study

OR: True odds ratio of the outcome variable per standard deviation of the exposure variable

R^2^: Proportion of variance explained for the association between the SNP or allele score

and the exposure variable
